# Around the collagen triple helix: an introduction to studying associated genetic and acquired diseases

**DOI:** 10.1016/j.matbio.2025.07.003

**Published:** 2025-07-07

**Authors:** Sergei P. Boudko

**Affiliations:** a Division of Nephrology and Hypertension, Department of Medicine, Vanderbilt University Medical Center, Nashville, TN, 37232; b Department of Biochemistry, Vanderbilt University, Nashville, TN, 37232; c AspirnautTM Program, Vanderbilt University Medical Center, Nashville, TN, 37232; d Center for Matrix Biology, Vanderbilt University Medical Center; Nashville, TN, 37232

**Keywords:** Collagen triple helix, folding, stability, mutation, peptide, therapy

## Abstract

The triple helix structure of collagen is the most abundant motif found in our bodies. It is believed to have emerged during the transition from unicellular to multicellular animal organisms, known as metazoans, and has evolved into various proteins that contribute to the development and function of diverse animal tissues, organs, and systems. Once synthesized, these collagenous proteins undergo post-translational modifications and proper folding inside the cell, after which they primarily function outside the cell. Over 80 collagenous proteins are categorized into two main groups: collagens and collagen-like proteins. However, the distinction between these groups is not clearly defined. Within these categories, there are various types of proteins, including soluble proteins, transmembrane proteins, and those that form the extracellular matrix. Multiple genetic diseases highlight the significance of collagenous proteins, which can be affected by defects in their primary structure, post-translational modifications, or complete loss. While fixing the gene defect may seem like a straightforward solution, we currently lack the capability to do so. Moreover, acquired diseases caused or accompanied by adverse processes in the collagen triple helix are generally not suitable for gene therapy at all. Understanding the pathogenicity of a defective polypeptide chain can provide valuable insights into strategies for mitigating negative consequences for both genetic and acquired diseases. This review highlights the current state of research in the collagen triple helix field, offering insights into how to study specific defects and deepen our understanding of their underlying pathogenic mechanisms.

## Introduction

The collagen triple helix has always been and remains to be one of the most mysterious protein motifs [1]. The triple helix had been a key player in the evolution of the multicellular animal species [2–5]. Due to unique physical and chemical properties it provides mechanical strength, resistance to harsh conditions, and at the same time it signals cells and provides scaffolding for building extracellular matrix [6–13].

The synthesis, post-translational modifications, folding, secretion, and assembly of collagen and collagen-like proteins depend on several machineries reviewed elsewhere [14–17]. This review focuses on the triple helix as a target for genetic and acquired pathologies, as well as the concepts related to studying and developing new therapies.

### Classification of collagens and collagen-like proteins

“Collagen” is a protein that produces gelatin when boiled, with its name derived from the Greek word meaning “glue-producing” [18]. The GXY repeat unit is a well-known characteristic of the collagen sequence, where every third amino acid is glycine (Gly), while proline (Pro) and hydroxyproline (Hyp) are commonly found in the X and Y positions. This understanding was not clear before the advent of sequencing and structural analysis techniques. Over a century ago, Dakin reliably determined the amino acid composition of gelatin [19], which made collagen one of the most studied proteins of that time. Subsequent analyses of gelatin and collagen composition by Atkin, along with X-ray diffraction studies conducted by Astbury, collectively led to the idea about the GXY pattern [20].

The first experimental indication of the GXY pattern likely came from Greenberg *et al* [21], who conducted sequential Edman degradation on mixtures of collagenase peptides derived from carp collagen. They discovered glycine in both the first and fourth positions of the peptide sequences. The definitive confirmation of the GXY pattern was obtained by Edman sequencing of a specific collagen peptide, α1-CB2, which was purified after cyanogen bromide cleavage by Bornstein [22]. This was followed by extensive sequencing of additional collagen peptides by different groups demonstrating the universality of the GXY rule.

**Fig. 1.**
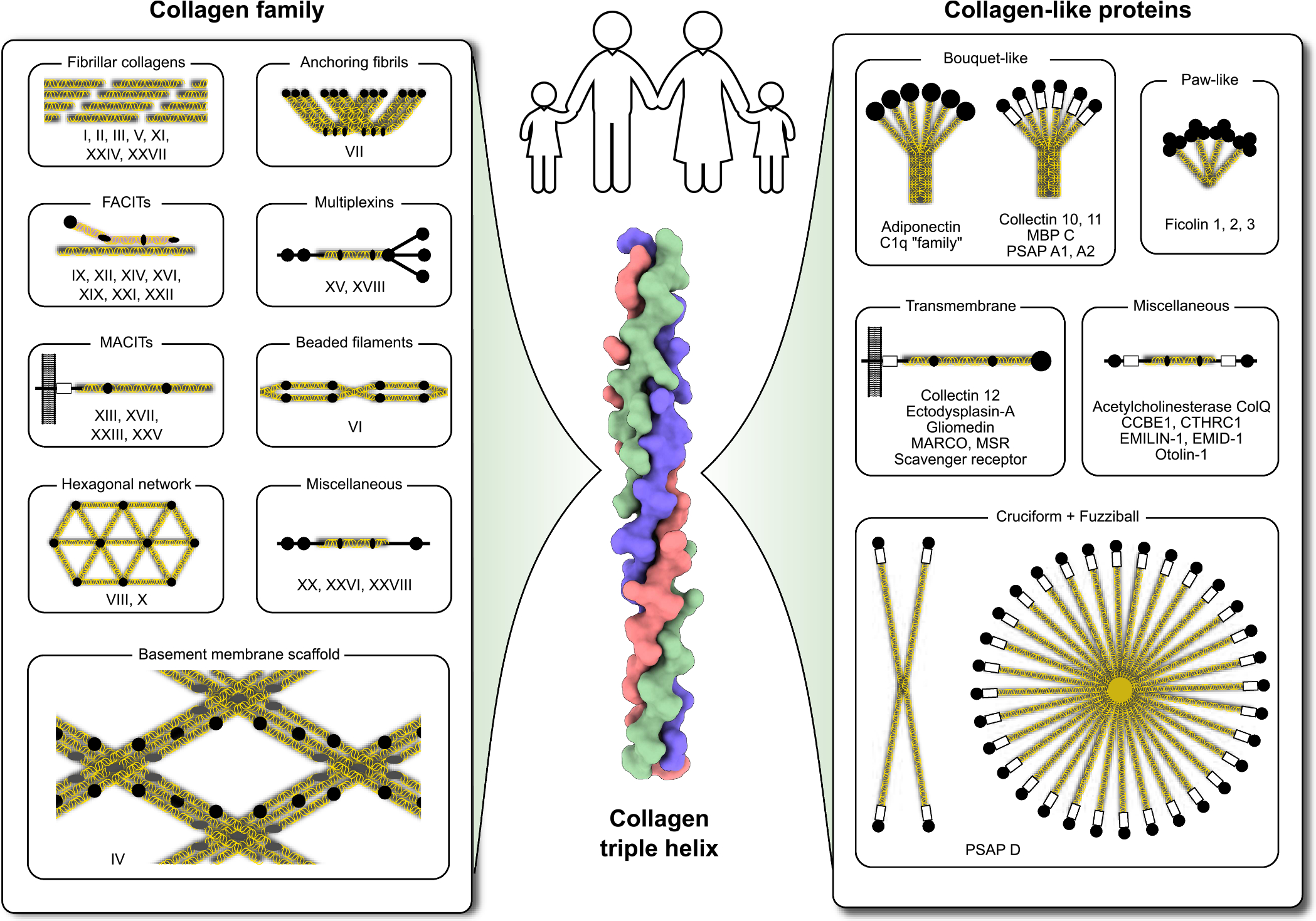
Collagens and collagen-like proteins. All proteins that contain the collagen triple helix are categorized into two families: “ collagens” (or collagens) and “collagen-like proteins.” The distinction between these two families is not entirely clear, and the classification of some proteins is still debated. However, a key difference is that collagens can be deposited as a matrix, while collagen-like proteins are primarily soluble in nature. Each family is further divided into classes based on the structural organization of the proteins and their roles. This classification can also be controversial, especially for collagen-like proteins, as the solubility and structural organization of many of these proteins remain unknown. Collagens are assigned a type number using Roman numerals from I to XXVIII, which generally reflects the order of their discovery and their resemblance to known types at that time. All 44 assignments for collagens and 39 for collagen-like proteins, along with additional information, can be found in Supplemental Figures 1 and 2. Silhouettes of humans, organs, and tissues in this and subsequent figures are obtained from SVG Repo.

Collagen was originally known simply as collagen until the discovery of a different type by Miller and Matukas in 1969 [23], specifically type II collagen found in cartilage. This marked the beginning of the collagen family. Since then, a total of 28 types of collagen have been identified (Fig. 1), with the most recent type, XXVIII, discovered in 2006 [24]. While collagens contain sequences of various other domains, the GXY repeat is a defining characteristic of all collagen types. Classical collagens are regarded as proteins that form the extracellular matrix. However, the GXY pattern has also been identified in a range of soluble proteins, which are referred to as collagen-like proteins (Fig. 1). Collagens and collagen-like proteins are further divided into classes based on their suprastructural organization (Fig. 1).

To create a comprehensive list of all collagens and collagen-like proteins in humans, all available protein sequences from UniProt (https://www.uniprot.org/) were re-analyzed to identify GXY repeats. How many repeats are necessary to guarantee the formation of a stable triple helix? Minicollagens found in Cnidarians contain 12–16 GXY repeats, which are believed to form the shortest stable triple helices [25]. However, based on our experience with synthetic collagen peptides, even fewer GXY repeats can maintain stability. Reliable and stable collagen triple helices can be achieved with just 9–10 repeats when using natural amino acids [26,27]. Only sequences with n=9 or more continuous (GXY)_n_G repeats were included and manually curated to exclude GGG and GGX patterns. Except for one protein, all identified proteins either contained a signal peptide for secretion or a type II transmembrane domain, with all GXY sequences located at the C-terminal (outside) end of the molecule. The exception was the pre-mRNA 3′ end processing protein WDR33 (UniProt code: Q9C0J8, isoform 1), which is located in the nucleus and contains a continuous stretch of 23 GXY tripeptide units. Further analysis using the AlphaFold3 prediction server did not yield conclusive results [28], leading to the exclusion of this protein from the list. It is important to note that AlphaFold3 has significantly improved upon AlphaFold2, as it successfully predicts the structure of a model featuring a hexamer of triple-helical trimers from the C1q complex [29]. Additionally, AlphaFold3 identifies the presence of a C-terminal trimerization domain in the Collagen triple helix repeat-containing protein 1 (Fig. S2). This domain is structurally homologous to that of the secreted chlamydial protein PGP3 and cannot be predicted using conserved domain prediction algorithms [30].

Finally, 44 collagens and 39 collagen-like proteins were identified. Collagen identification follows a recognized classification of matrisome proteins [31] and aligns with UniProt assignments. Other proteins are categorized as collagen-like proteins. This is probably the most comprehensive list of human collagens and collagen-like proteins to date. A schematic representation of each protein with its UniProt identifier is provided in Figures S1 (collagens) and S2 (collagen-like). A full list of collagen-like proteins is shown in Table. Interestingly, ficolin-3 was found to have the shortest single (GXY)_n_G pattern, consisting of only n=10 repeats (Fig. S2).

It is important to note that the classification is likely to be refined significantly as we gain more knowledge about these proteins. Notably, collagen XVI and EMI domain-containing protein 1 have similar domain structures (see Figs. S1 and S2), indicating that they may belong to the same group. It also remains unclear what differentiates MACIT collagens from transmembrane collagen-like proteins.

### The triple helix

Collagen captured the interest of many distinguished chemists and physicists in the mid-20^th^ century, leading to a competition to develop an atomic model of this protein. Ramachandran and Kartha from Madras emerged victorious in this race, proposing the coiled-coil triple helical structure of collagen for the first time [32]. The accurate atomic structures of the collagen triple helix were ultimately revealed through the first crystal structures solved by Okuyama *et al* [33] and Bella *et al* [34]. The detailed analysis of the triple helix geometries is provided by Okuyama *et al* [35,36]. Three left-handed helices combine to form a right-handed superhelix, featuring two distinct helical symmetries: 7/2 [37] and 10/3 [38,39]. This coexistence of both symmetries was demonstrated in a fragment of collagen derived from a natural sequence [40]. The 10/3 symmetry is particularly associated with regions that are deficient in imino acids (proline and hydroxyproline) [36,40]. The typical distribution of proline residues shows that regions lacking imino acids, characterized by three or more proline-free GXY units, occupy significantly fewer sequences (Fig. 3). Therefore, the 7/2 symmetry is anticipated to prevail.

Recent study has revealed an unexpected structure of the C1q complement fragment, showcasing a novel type of collagen triple helix that lacks the typical superhelical twist [29]. This finding suggests that collagen and collagen-like assemblies possess a much more diverse conformational landscape than previously recognized. This is particularly likely in suprastructural assemblies of triple helices. Our understanding of the functionality of collagenous scaffolds is significantly limited, largely due to the scarcity of experimental structural data regarding the packing of triple helices. Even the understanding of the packing of the most abundant and most studied collagen I fibrils remains obscure [41].

The triple helix structure brings side chains of residues in X and Y positions fully exposed to the solvent that allows to accommodate any of 20 regular amino acids and even their post-translational modifications, even the quite bulky ones (Fig. 4). Such an open architecture provides an opportunity to create highly informative sites along the triple helix, which can also help in discovering artificial sites not restricted to collagen-binding proteins [42]. Indeed, 2/3 of residues are ready to interact with the world (Fig 4C). On the other hand, stability of the triple helix requires a fee, sufficient amount of stabilizing factors. Two major factors are presence of imino acids (proline and hydroxyproline) and specific arrangement of oppositely charged residues [43]. Indeed, the most frequent residues are proline in X and Y positions. Hydroxylation of proline in Y position additionally increases the triple helix stability [44–46]. The second most frequent residues are glutamate (negative) in X and lysine (positive) in Y positions of either collagens or collagen-like proteins (Fig. 5A and B). Interestingly, in bacterial collagen-like proteins frequency of proline in Y position is significantly lower and frequencies of charged and polar residues are much elevated (Fig. 5C). Due to the lack of proline hydroxylation machinery in bacteria, the major stabilizing factor becomes the ionic and polar interactions [47–49].

### Diseases

Genetic variants that directly impact the triple helical structure can be highly detrimental. These include deletions, insertions and deletions (indels), the generation of premature stop codons, and missense (or point) mutations that result in an amino acid change. Although non-missense mutations can also cause significant and serious diseases, their pathological mechanisms typically lead to intracellular damage or complete loss of the protein, which are not directly associated with the functionality of the triple helix. Missense mutations are categorized into glycine and non-glycine (or X and Y) mutations, with over 40 genetic diseases linked to this type of mutation, affecting nearly every organ and tissue (Fig. 6).

**Fig. 2.**
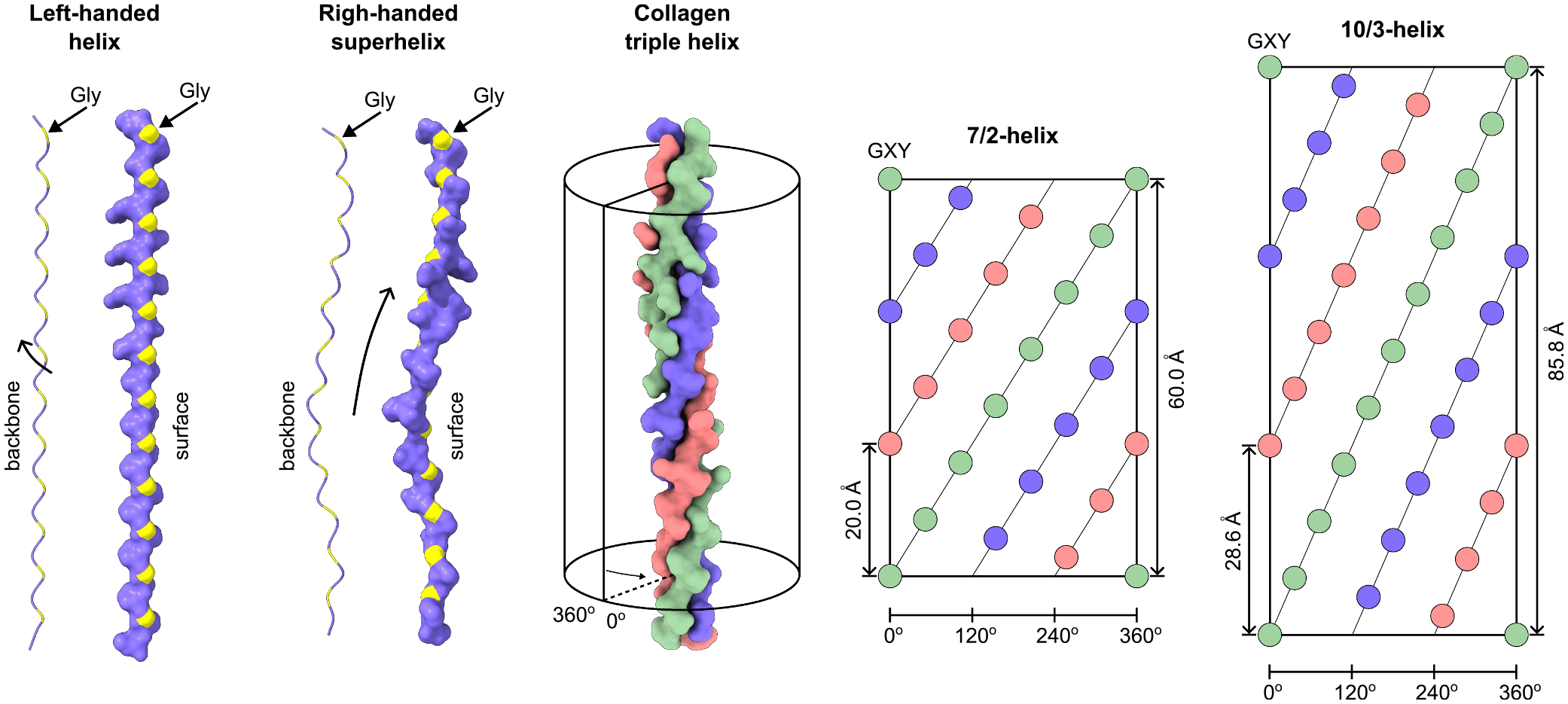
Geometry of the collagen triple helix. The geometry of the collagen triple helix shows that each chain typically exhibits a left-handed helical structure combined with a right-handed superhelical twist. Two primary symmetries have been modelled and later experimentally confirmed, known as the 7/2-helix and the 10/3-helix. The 7/2-helix is characteristic of sequences rich in imino acids, *i.e*., proline and hydroxyproline residues. In contrast, the 10/3-helix is associated with regions that are low in imino acids, lacking proline and hydroxyproline residues.

**Fig. 3.**
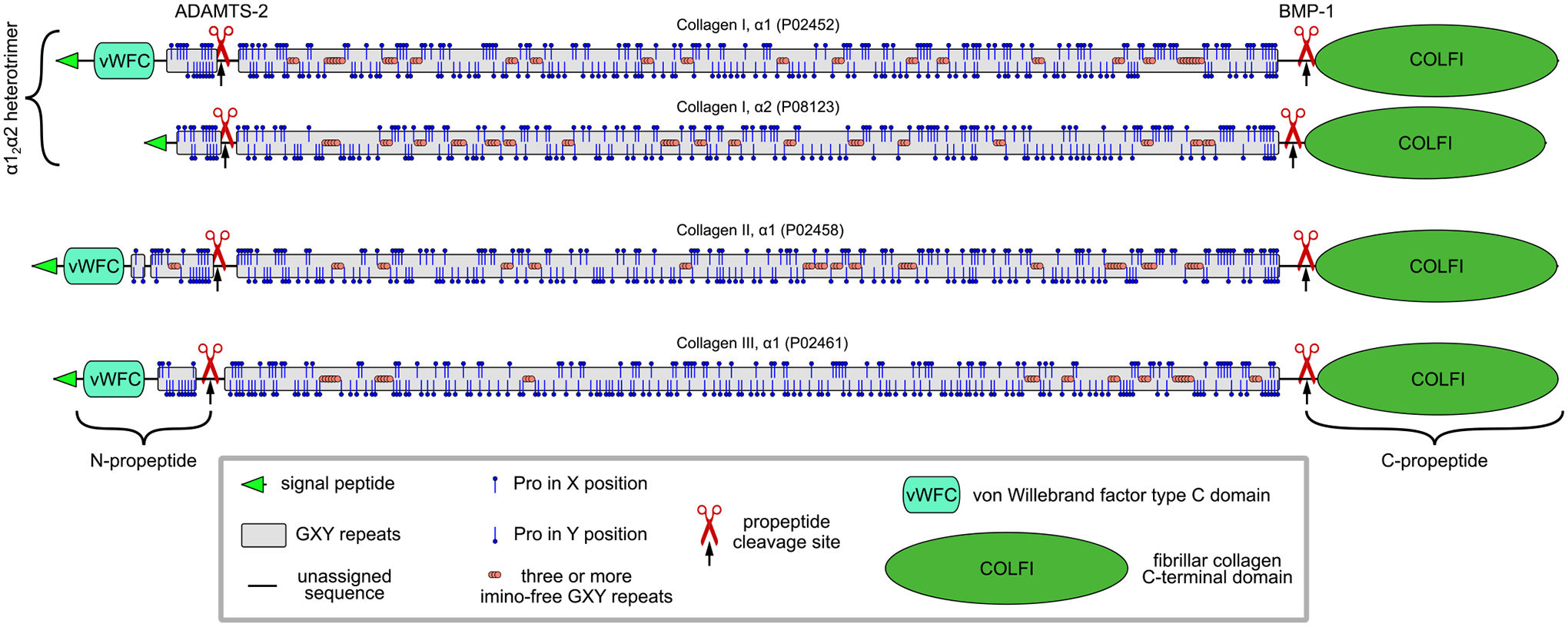
Distribution of proline residues and regions that lack imino acids in collagens I to III. Mature collagen sequences are those devoid of N- and C-propeptides, which are removed by specific proteases. Proline residues in X (up pins) and Y (down pins) positions are not evenly distributed along the triple helix. There are regions that are mixed, predominantly contain X- or Y-positioned prolines, or free of proline residues. Regions containing more than two proline-free repeating GXY units are highlighted with orange circles. These areas are expected to form the 10/3-helix structure in homotrimeric types of collagens, such as types II and III. In collagen I, which is composed of two α1 chains and one α2 chain, it is important to consider the compositional match of these regions.

**Fig. 4.**
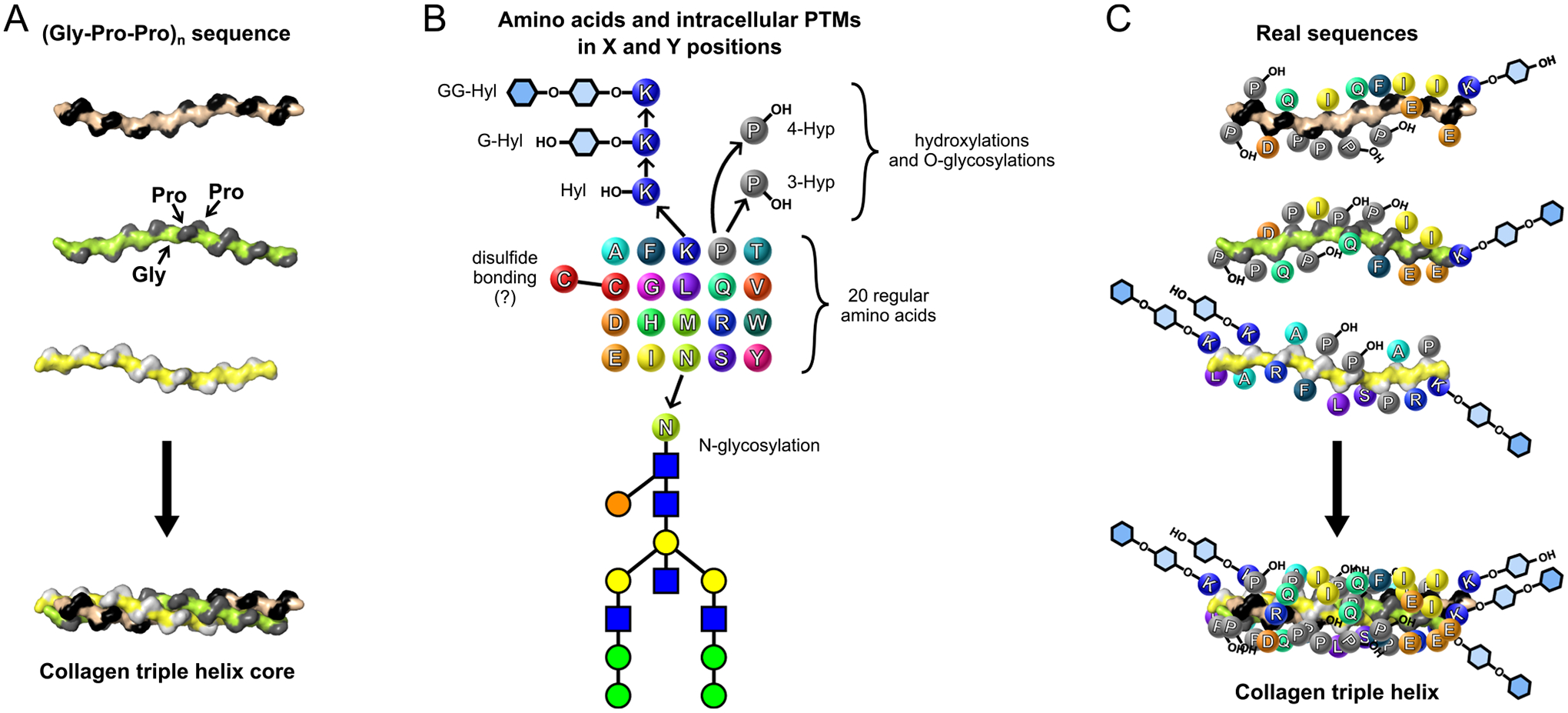
A wealth of information and intracellular post-translational modifications to build functional triple helices. (A) Gly-Pro-Pro tripeptide units are key structural elements of the triple helix. While proline and their modifications are important stabilizing residues, the only requirement for building the triple helical structure is the presence of glycine in every third position. Glycine being a residue with the smallest side chain (a single hydrogen atom) is the only one that fits into the triple helical structure. Side chains in the X and Y positions are solvent-exposed and can fit any amino acid. (B) Residues in X and Y positions are often not prolines and their modifications. Any of the regular 20 amino acids has been found in these positions. In addition, there are two post-translational modifications (PTMs) of proline residue into 4-hydroxyproline (4-Hyp) and 3-hydroxyroline (3-Hyp); three PTMs of lysine residue into hydroxylysine (Hyl), galactosyl-hydroxylysine (G-Hyl), and glucosyl-galactosyl-hydroxylysine (GG-Hyl); N-glycosylation of asparagine; and potential disulfide bond formation between cysteine residues under oxidative environment of the endoplasmic reticulum. (C) Since residues in the X and Y positions are all solvent-exposed, all of them (or 2/3 of a sequence) are readily available for interactions with other molecules. Such a unique organization of the collagen triple helix allows the combination of tensile strength, proteolytic resistance, and chemical stability with the ability to present a lot of information on the surface of the molecule. In contrast, globular proteins bury a significant portion of residues to build their core structure.

**Fig. 5.**
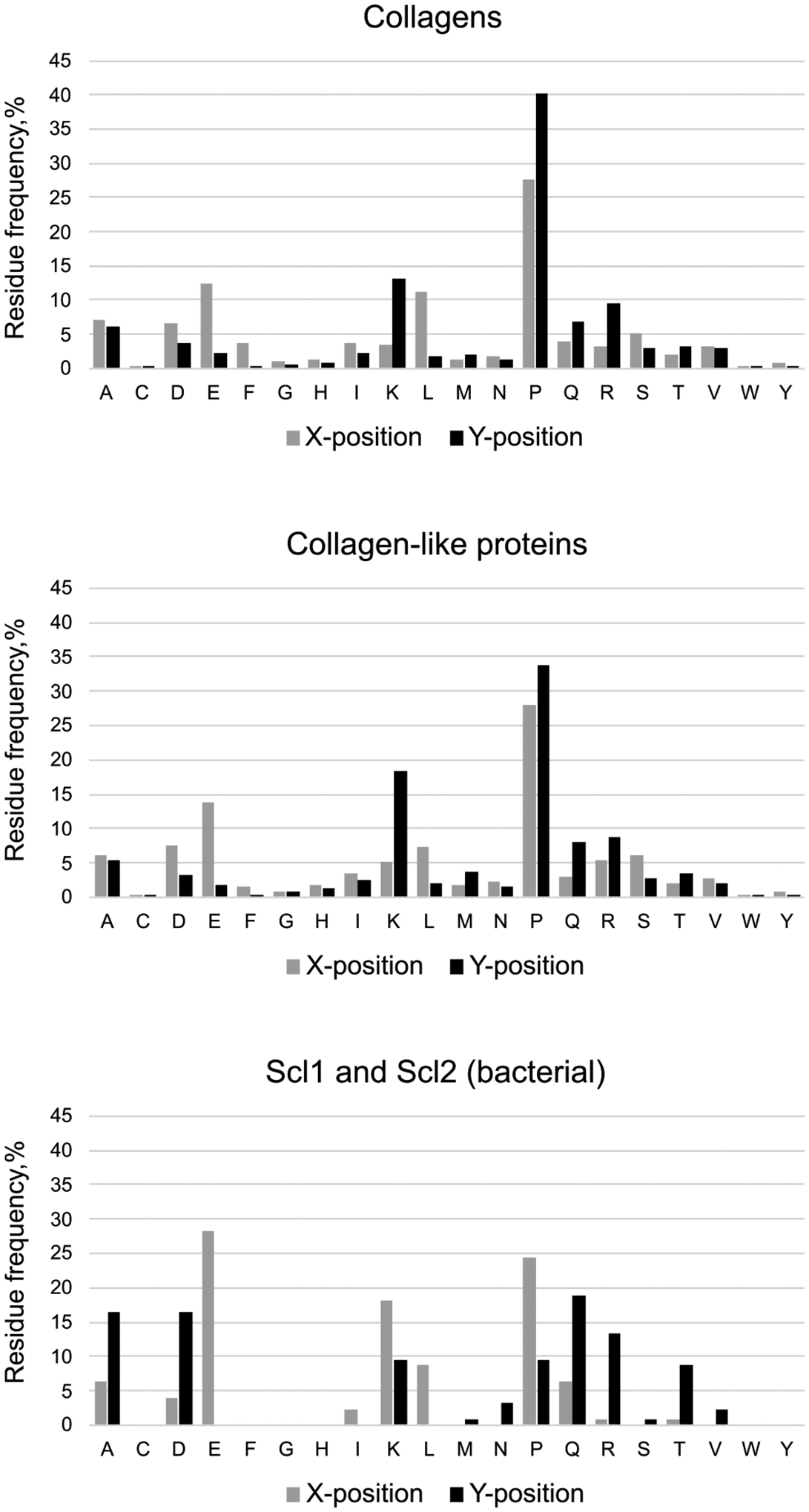
Distribution of residues in X and Y positions. (A) Frequency of residues found in human collagens. (B) Frequency of residues found in human collagen-like proteins. (C) Frequency of residues found in bacterial collagen-like proteins Scl1 and Scl2 from group A *Streptococcus* [198]. Frequencies were normalized to 100% for each position (X and Y).

**Fig. 6.**
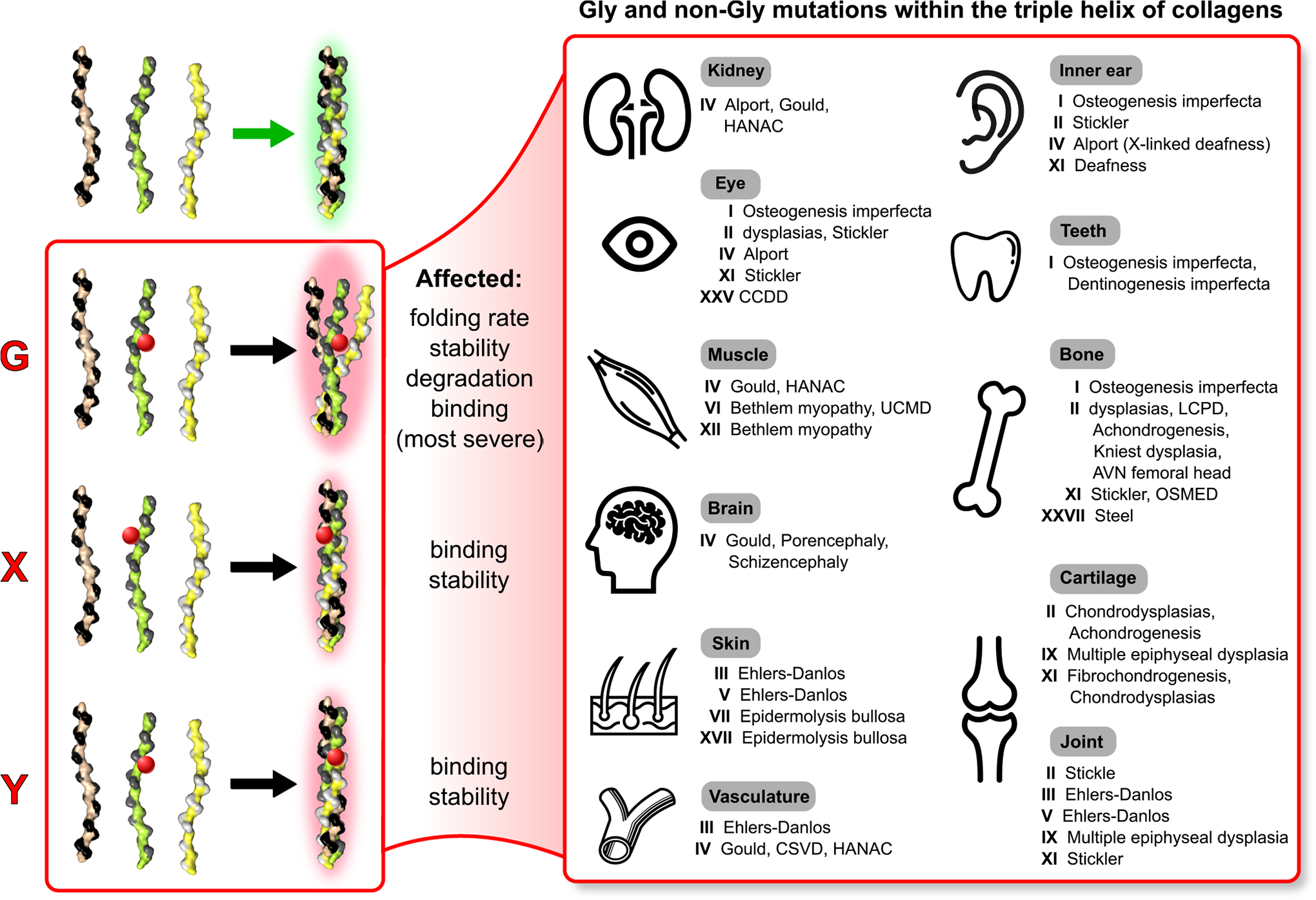
Missense mutations in the Gly, X, or Y positions of the triple helix can cause a wide range of diseases that affect different organs and tissues. Glycine (Gly) substitutions cause the most significant disruption to the triple helical structure, often resulting in more severe phenotypes. The effects of Gly mutations can lead to multiple problems, including delays in folding at the mutation site, which can result in excessive modification of the unfolded regions of the triple helix. Additionally, these mutations can significantly destabilize the structure, leading to increased degradation, and can disturb nearby binding sites for ligands and extracellular molecules. In contrast, mutations at X and Y positions typically have moderate or mild effects, as these positions are generally more tolerant to substitutions. However, these residues may still play a crucial role in local stabilizing interactions with residues from the same chain or neighboring chains in the triple helix. Disrupting these interactions can undermine the stability of the triple helical structure. Furthermore, residues in the X and Y positions may be involved in binding to ligands and receptors or in forming superstructural complexes with other molecules, contributing to the functionality of the matrix. Overall, missense mutations in nearly all types of collagen lead to a wide range of disorders that impact various organs and tissues (right panel).

Glycine substitutions for any other amino acid usually result in the most severe phenotypes, as they violate the steric constraints necessary for the three chains to properly associate and form the super-coiled helix (Fig. 2). The immediate consequence of such mutations is a significant delay in the folding of the triple helix [50,51], which follows a zipper-like mechanism [52]. The specific nature of the substitution is also crucial, as bulkier residues tend to have a more pronounced effect [51]. These delays lead to post-translational overmodifications of other residues following the mutation, as they remain accessible to the extensive collagen processing machinery for extended period [53]. Furthermore, the altered geometry of the resulting structure makes it more flexible, less stable, and more susceptible to degradation. If the mutation occurs near an important binding site, whether for constructing suprastructures or for cell signaling receptors, the functionality of that site will also be negatively affected.

Genetic variants found in positions X and Y are typically less harmful because they are not anticipated to hinder the folding process. However, their effects may include reduced stability, resulting from disruptions in local interactions—both intra-chain and inter-chain—between the side chains of neighboring residues. Additionally, these variants could potentially disturb any binding sites they are associated with.

Acquired changes that are non-genetic are very complex. Notably, both excessive collagen deposition associated with fibrosis or sclerosis and the excessive degradation of collagen scaffolds are areas of active research described in the section on Emerging therapies. It is important to note though that under chronic inflammation, the quality of the triple helix differs significantly from normal conditions, as it becomes excessively modified [54]. The thermal stability of the triple helix which is linked to post-translational modifications is a crucial factor that will be further explored in the section on Critical features.

**Fig. 7.**
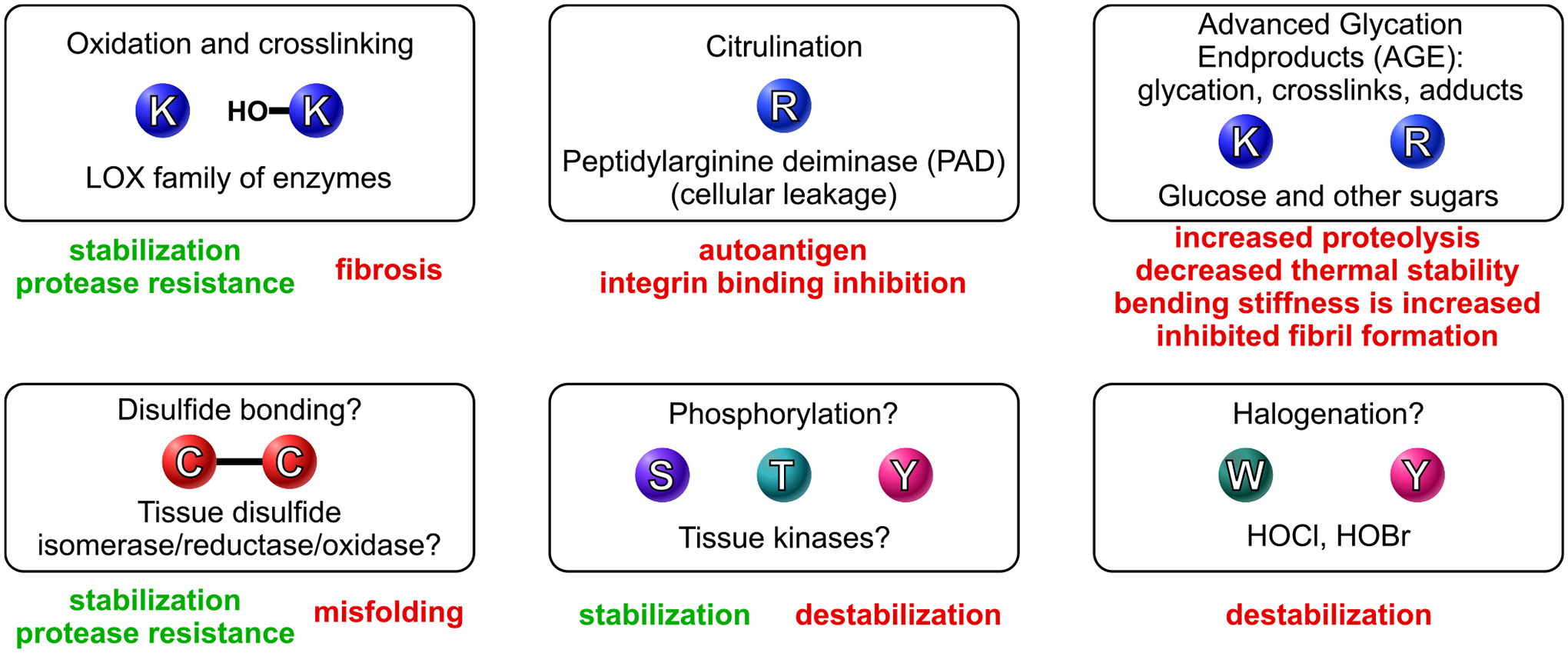
Extracellular modifications of the triple helix. Once secreted into the extracellular space, some residues undergo additional modifications. Key modifications include the oxidation of lysine and hydroxylysine residues, leading to crosslinking, as well as the oxidation of cysteines and the formation of disulfide bonds. These processes are critical for maturation into functional scaffolds, fibrils, and other suprastructures. While the phosphorylation of the triple helix remains an unexplored area, it may also play a significant role. In contrast, other modifications, such as the citrullination of arginine residues and the glycosylation of lysine and arginine residues (which lead to the formation of crosslinks and adducts), as well as the halogenation of tryptophan and tyrosine residues, often have detrimental effects.

Apart from significant functional modifications that occur to the triple helical residues outside the cell—such as the oxidation and crosslinking of lysine and hydroxylysine residues, as well as the formation of disulfide bonds—several other modifications have been reported in collagens due to their very slow turnover rates (Fig. 7). The *in vitro* phosphorylation of serine and threonine residues in the triple helix [55,56], which has yet to be confirmed in real tissues, may potentially offer stabilizing benefits [55] or indicate unknown signaling functions [57]. Halogenation has also been reported in collagens [58–60], but it remains uncertain whether the same modifications can accumulate in the triple helical sequences. Notably, citrullination and the formation of advanced glycation end (AGE) products are well-documented adverse modifications within the triple helix [61,62]. Citrullination of collagen II, and potentially collagen IX, occurs when peptidyl arginine deiminases leak from cells [63], which can trigger an autoimmune response in rheumatoid arthritis [61]. One study also reported that this process can inhibit integrin binding. AGEing leads to various issues affecting collagen fibrils and individual triple helices [62,64].

### Critical features of the triple helix

When studying collagen *in vivo* or *in vitro* it is important to keep in mind several critical features of the triple helix to avoid common mistakes that may lead to unexpected results and incorrect conclusions:

#### Marginal stability.

Nature adjusts the hydroxyproline content in collagen to ensure that the melting temperature of its triple helical domains is a few degrees below body temperature, rather than above it [65]. In fibers or other suprastructural assemblies, collagen helices can melt and refold locally when necessary, providing elasticity and allowing for turnover within these structures [65]. From an evolutionary perspective, the balance of stabilizing factors including post-translational modifications and informational load (binding sites) has been finely tuned to maintain both functionality and stability [66–73]. When collagen is unstable at the protomer level, it requires special handling both inside the cell and during its incorporation into the appropriate suprastructures that further stabilize it. A collagen-specific chaperone known as heat-shock protein 47 (HSP47) plays a crucial role in this process [74–76]. In cases of inflammation due to increased temperature, collagen can become overmodified and partially compensate thermal instability [54]. When working with isolated collagen molecules, it is essential to take special care to prevent them from unfolding.

#### Don’t study scurvy unless it is the specific subject of your research.

Ascorbate, commonly referred to as vitamin C, is essential for collagen synthesis, stability, and secretion [77,78]. It acts as a cofactor for enzymes involved in the hydroxylation of proline and lysine, which are critical steps in collagen production. Without adequate levels of ascorbate, these hydroxylation reactions cannot occur properly, which can impair collagen formation and result in conditions such as scurvy. Ascorbate must be regularly supplemented to the culture media due to its instability at neutral pH. It is never supplied in commercially available media.

#### Cysteines in the triple helix.

**Fig. 8.**
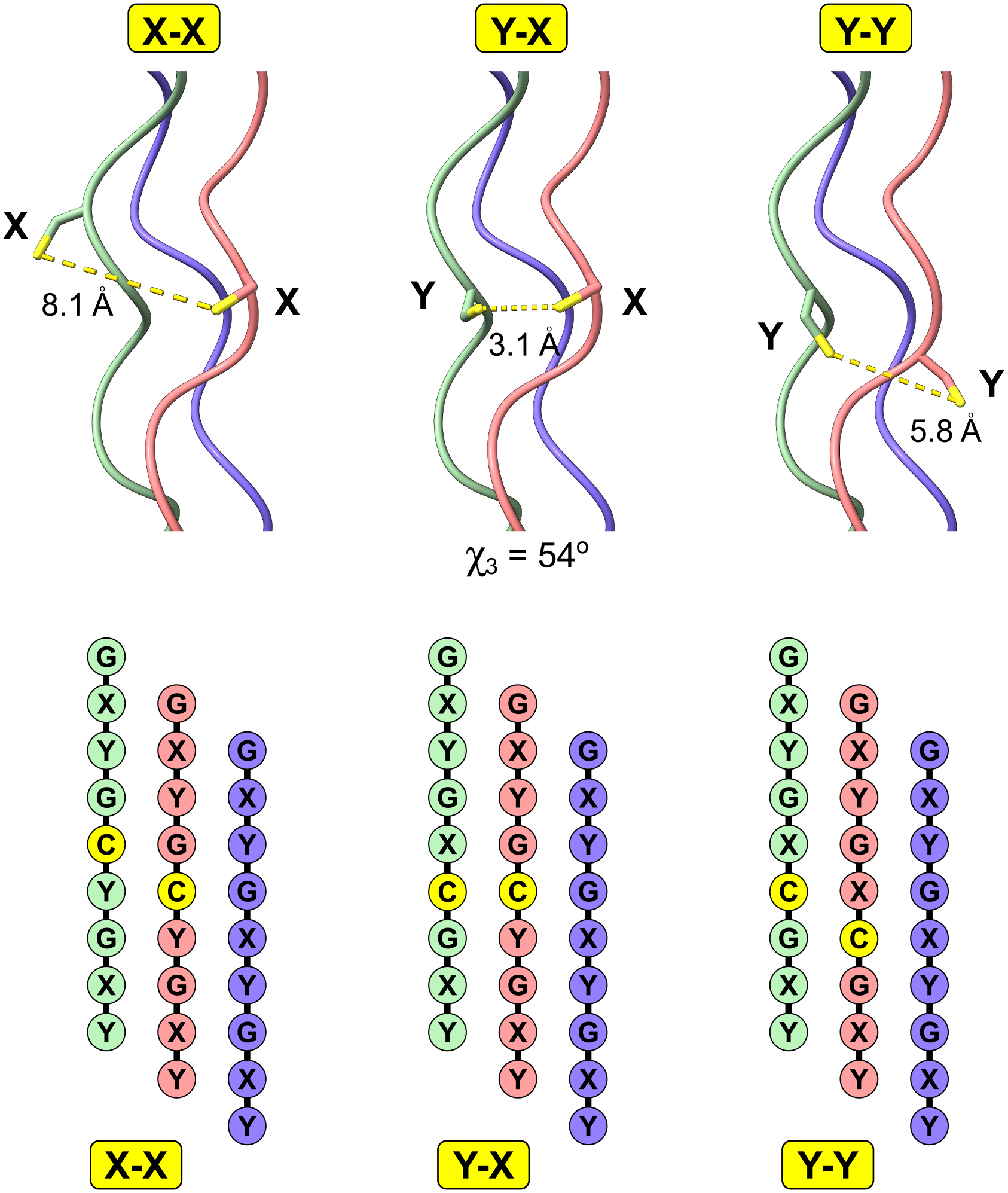
Cysteines in the triple helix. Cysteines are particularly interesting because collagens and collagen-like proteins operate under oxidative conditions, and all cysteines in the X and Y positions are exposed to solvent. Therefore, they must undergo oxidative modifications. Is it possible for cysteines from two adjacent chains to form an interchain disulfide bond? When both cysteines are located in either the X or Y positions, they are too far apart to form a disulfide bond. However, there is a special case where one cysteine is in the Y position of one chain and the other is in the X position. In this scenario, there is a slight possibility for them to form a disulfide bond, but this would require some structural changes. The distance between the sulfhydryl groups remains below the required threshold for bonding—the optimal distance is 2.1 Å, while the PDB cutoff is 3.0 Å. Additionally, the χ3 dihedral angle deviates from the ideal values, measuring at −87^◦^ and +97^◦^ [199].

Due to the oxidative environment in the endoplasmic reticulum and the extracellular space where collagen and collagen-like proteins function, the sulfhydryl group of cysteine is likely to oxidize. Given the structural organization of the triple helix, cysteines typically cannot form disulfide bonds within the same helix without compromising the overall geometry (Fig. 8). However, there is one special case in which a cysteine positioned at the Y site can form a disulfide bond with a cysteine positioned at the X site within staggered chains (see Fig. 8B). This situation allows for some chain adjustments that could facilitate interchain disulfide bond formation.

For human sequences, there are only two instances where this occurs. The first is the α345 isoform of collagen IV [79], which is composed of three chains: α3, α4, and α5. In this case, one cysteine is located in the Y position of the α3 chain and another in the X position of the α4 chain, both found within the last C-terminal triple-helical fragment (see Fig. S1). The placement of the X cysteine near the border of the GXY fragment may promote chain adjustments to achieve a better geometric fit for disulfide bond formation. The second instance is found in otolin-1, where two cysteines are located within the same GXY repeat. Out of the six cysteines present, four can form two disulfide bonds, while the remaining two must interact with other molecules. In summary, most cysteines within the triple helix either participate in the formation of disulfide bonds with other molecules or become oxidized to non-reactive forms.

Consequently, special care should be taken when handling these cysteines, including cysteine mutations.

#### Insolubility.

Collagens typically self-associate into higher-order suprastructures. Extracting all collagen from tissues or *in vitro* cultures is often difficult and sometimes impossible. Always verify what remains in the pelleted fraction.

#### Asymmetry.

**Fig. 9.**
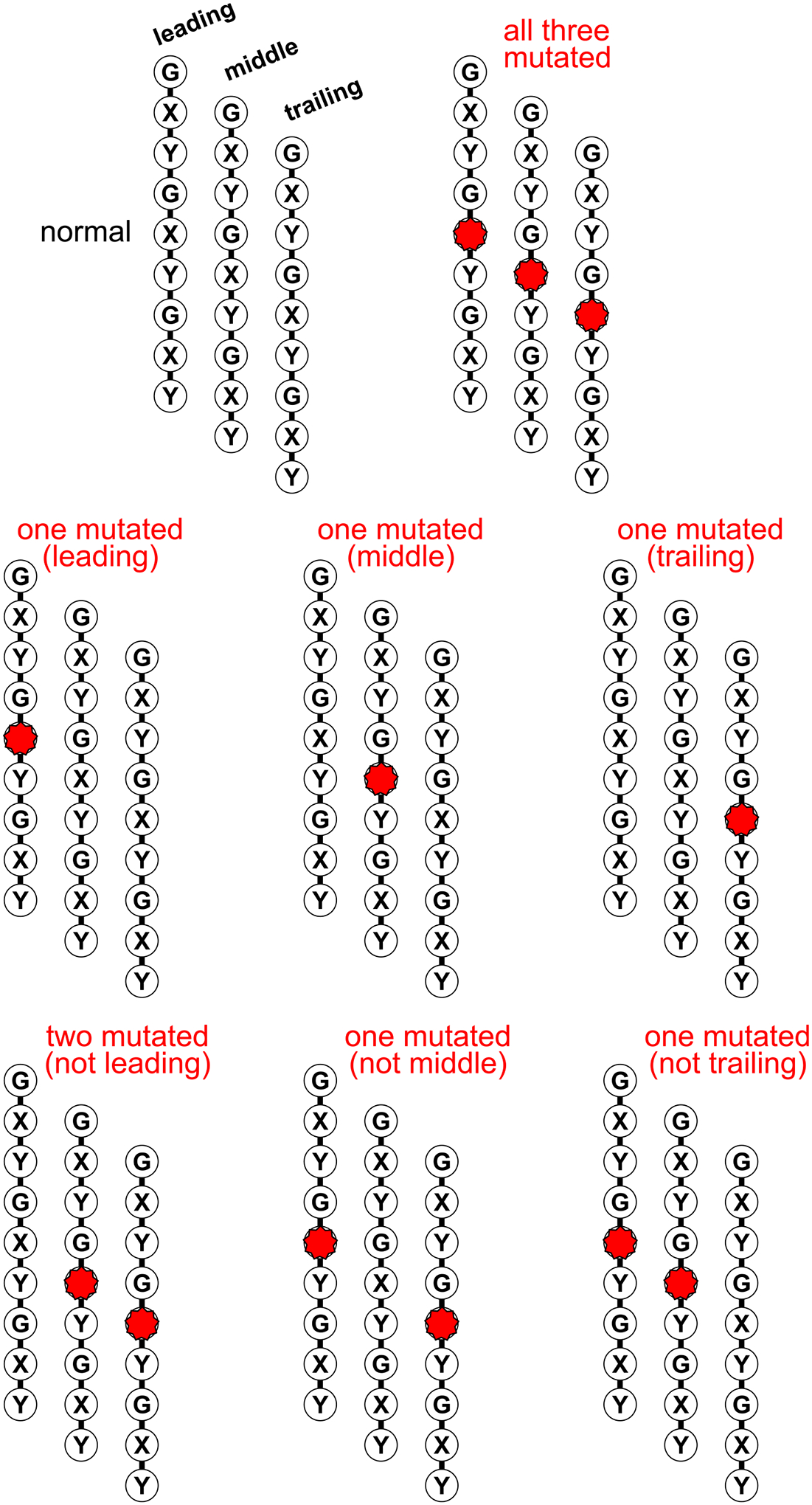
Multiple structural variants of the triple helix with a mutation. Due to asymmetric nature of the triple helix each chain is structurally unique even in the homotrimeric type. A point mutation in one allele can result in eight possible variants of the triple helix. This situation highlights the need for specialized tools to study each unique structural variant effectively.

The triple helix structure is asymmetric, consisting of three chains that follow a specific arrangement. These chains include a leading chain, a middle chain, and a trailing chain, where each is shifted by one residue in relation to the others. As a result, a single point mutation can potentially create up to eight different structural variations (Fig. 9). Investigating point mutations in collagen and collagen-like proteins necessitates special techniques detailed in the Tools section.

### Key tools to study the triple helix

#### Synthetic peptides.

Collagens are large, insoluble proteins that are challenging to extract from tissues and work with. The development of peptide synthesis methods has enabled researchers to study short, soluble fragments known as triple-helical peptides (THPs). These have been successfully used as models for collagen since the 1960s [26,27]. Over time, the focus of THP-based research has broadened from investigating the structural determinants of collagen to screening binding affinities and exploring the effects of mutations [26,80–83].

This technology has also allowed the creation of libraries of synthetic peptides that cover the entire sequences of collagens II and III [84]. This allowed for precise mapping of hundreds of binding sites along collagen sequences in relation to various receptors and proteins. Peptide synthesis enables precise control over chemical composition, ensuring the incorporation of specific amino and imino acid modifications. Additionally, it allows for the use of unnatural modifications and the introduction of specific crosslinks to enhance stability and regulate chain composition in heterotrimeric peptides [85–92]. However, a limitation of this method is that the size of the fragments generally does not exceed 30–50 residues.

#### Recombinant production.

The recombinant production of triple helical fragments addresses the limitations of peptide synthesis in terms of length but introduces other challenges, such as post-translational modifications and chain alignment. Typically, THPs can self-assemble into properly aligned structures due to their short length. Even slight misalignments of a few GXY tripeptide units can become energetically unfavorable, leading these misaligned structures to be less populated.

For longer chains, these misalignments can cause issues known as the gelation process. In this case, individual collagen chains form multiple local triple-helical associations with one another, resulting in high-order aggregates or even a continuous gel. The inclusion of collagen-specific trimerization domains [93,94] effectively addresses this issue. Examples of suitable trimerization domains include those found in collagen types IX, XV, XVIII, and XIX [95–98], as well as a collagen-unrelated foldon domain from the T4 phage [99–102]. These domains are small, ranging from 30 to 54 residues long, and they can be efficiently expressed even in bacterial systems.

When post-translational modifications are necessary, suitable expression systems must be employed [103]. These systems can be further optimized with components that facilitate collagen post-translational modifications (PTMs) to ensure that the modifications are adequately executed.

#### Folding assays.

While synthetic and short recombinant peptides offer some insights into the folding of triple helices, they do not provide a natural context for this process, which involves a zipper-like propagation [52]. The folding of short peptides that are not crosslinked or not trimerized relies heavily on the helix nucleation process, which must accurately align three separate chains [104]. This alignment is a complex event that obscures the subsequent helix propagation. Even in cases where short peptides are crosslinked or trimerized, destabilizing mutations can significantly hinder helix propagation.

To address this issue, it is necessary to use constructs that are sufficiently long, typically between 100 and 300 residues [51,105]. This length helps to mitigate local destabilization caused by mutations, allowing helix propagation to be restored at a distance from the mutation site. Additionally, longer triple helices provide a more substantial signal for monitoring the folding process. These conditions are predominantly achievable through a recombinant approach.

#### Detection.

Whereas type- or protein-specific antibodies are invaluable tools in detecting collagens and collagen-like proteins in tissues, solid-phase assays, Western-blots, etc. their price, availability, and overall low specificity to the triple helical sequences, which usually have very low antigenicity, impose certain limitations in collagen studies. Fortunately, alternative ways emerged that specifically target the triple helical sequences.

The concept of collagen hybridizing peptides (CHPs) was introduced in 2005 [106]. It relies on the inherent tendency of peptides with the GXY repeats to self-assemble into a triple helix structure. A short and labeled peptide can bond with denatured strands of virtually any type of collagen under appropriate conditions. This technique has advanced significantly and is now widely used to detect denatured collagen in tissues and protein gels [107–115]. Among them there are various ready-to-use forms of CHPs, as well as chemically or light-activated versions with multiple reporters, which enable a broad range of applications in collagen research.

An alternative approach involves using the *Staphylococcal* collagen adhesin fragment known as CNA35, which specifically binds to the triple helical structure of collagen [116,117]. There are various CNA35 probes available, each labeled with different dyes and reporters [118–125]. This fragment is widely used for tissue staining in both *in vitro* and *in vivo* experiments. Recently, it has also been adapted for protein detection in Western blots [126].

Together, the universal applicability of CHP and CNA35 for the specific detection of triple helical sequences, along with their complementary abilities to detect either denatured (single chain) sequences via CHP or folded (triple helical) structures via CNA35, makes them essential reagents for any laboratory specializing in collagen research.

#### Composition and stagger control.

Whether you are interested in producing the heterotrimeric triple helix structure or a specific variant with a point mutation, it is essential to control the chain composition and the stagger (or register) of the chains. Both peptide synthesis and the recombinant production of collagenous fragments have developed corresponding tools for these purposes.

Several strategies have been developed for producing synthetic heterotrimeric collagen peptides. These include regio-selective chemistry for crosslinking three peptides using pairs of cysteines [85,86,127] and the complementary designs of host sequences that ensure the registered assembly of three or two different sequences, incorporating guest sequences of interest [128–132].

For recombinant production, the trimerization domain of collagen IX has been successfully utilized to produce both short and long collagenous fragments, approximately 200 residues in length [95,103,133]. The available crystal structure of the NC2 domain containing the collagenous sequences has confirmed the properties of heterotrimerization and register control [134].

### Emerging therapies

#### Crosslinking.

The tensile strength of the collagen triple helix is crucial for its mechanical performance, and additional crosslinking of collagen structures can further enhance this strength. However, the effectiveness of this reinforcement may vary under certain pathogenic conditions. One potential solution is the artificial crosslinking of an altered collagen matrix. A notable example of this is the crosslinking of corneal collagen for keratoconus using vitamin B2 (riboflavin), which is converted into reactive species through UV light exposure [135]. This process initiates collagen crosslinking, thereby strengthening collagen fibers in the cornea that are otherwise prone to mechanical deformation. While applying this technique to other tissues is problematic, it offers a promising concept that could potentially be adapted for treating other collagen-associated disorders with the successful development of targeted crosslinking methods using different chemical approaches.

#### Protein replacement.

The groundbreaking work in developing a novel protein replacement therapy in the field of collagen aims to find a cure for dystrophic epidermolysis bullosa. Researchers have successfully created a recombinant production platform to generate collagen VII, a frequent culprit of the disease [136–138]. This recombinant collagen can be injected subcutaneously and exhibits therapeutic effects [139–142]. This achievement should inspire other researchers to apply the same approach to other conditions caused by a deficiency of specific collagen isoforms.

An intriguing alternative for replacing missing or malfunctioning human collagens or collagen-like proteins is the use of bacterial collagen-like proteins, such as Scl2 from Streptococcus [143]. These proteins are easier to produce because they do not require the complex machinery needed for post-translational modifications. Additionally, they have evolved to evade the human immune response, making them a compatible material. If necessary, these proteins can be extended and modified to meet the specific functionality required for the type of protein being replaced, at least as a temporary emergency substitute.

#### Collagen peptides.

Collagen peptides, also known as collagen hybridizing peptides (CHP) or collagen mimetic peptides (CMP), have a strong tendency to adopt the triple helical structure of collagen. These peptides can bind to denatured collagen strands through a process called strand hybridization [114,144], which was discussed above in the section on Key features. CHP and CMP are self-targeting, allowing them to reach areas undergoing collagen remodeling, whether in normal conditions or in response to injury, inflammation, and fibrosis. Their potential applications include diagnostic imaging and drug delivery [145–151]. Additionally, these peptides are actively being researched as therapeutic agents that can help repair and restore unfolded sites within collagen suprastructures [152–158].

#### Targeting the triple helix.

Apart from collagen peptides that bind denatured strands of collagen there are different types of collagen-binding proteins ranging from matrix proteins to receptors and to bacterial adhesins. Their collagen binding domains (CBDs) can be used as vehicles to target collagenous suprastructures either for diagnostic or therapeutic purposes [159]. CBDs are derived from natural ligands of collagen, such as fibronectin, von Willebrand factor, placental growth factor, and collagenase [160–163]. CBDs have been utilized to enhance wound healing, support vascular repair, promote bone regeneration, treat tumors, deliver growth factors for tissue repair, and address developmental defects [160,164–167]. Furthermore, CNA35, which is commonly used for collagen detection and imaging, could potentially be utilized for targeted drug delivery.

#### Reducing collagen production, deposition, and turnover time.

There are several emerging strategies to address the issue of excessive collagen in conditions like fibrosis and sclerosis by influencing its production, deposition, and turnover time. One promising therapeutic target in fibrosis is HSP47, a collagen-specific chaperone [168–172]. Another potential approach is the inhibition of SPARC (secreted protein, acidic and rich in cysteine), which may serve as a useful strategy for collagen management [173]. Additionally, targeting collagen prolyl 4-hydroxylases is an appealing strategy, as 4-hydroxyproline residues are essential for maintaining the stability of the collagen triple helix [174,175].

Limiting collagen fibril formation is another avenue of exploration to reduce fibrosis [176,177]. Recent advances have been made in developing monoclonal antibodies aimed at blocking collagen fibrillogenesis in cases of post-traumatic arthrofibrosis [178–180] and in testing small molecules designed to inhibit fibril formation in idiopathic pulmonary fibrosis [181].

Collagen crosslinking appears to be crucial for prolonging the lifespan of deposited collagen structures. Therefore, inhibiting collagen crosslinking holds potential for reducing turnover time and alleviating fibrosis and sclerosis. Lysyl oxidase-like 2 (LOXL2), known to promote collagen crosslinking [182], has emerged as a promising target for decreasing fibrosis in cardiac, liver, lung, and renal tissues [183–188]. Inhibition of LOXL2 also has the potential to alleviate glomerulosclerosis and albuminuria in diabetic nephropathy [189]. Moreover, LOXL2 plays a significant role in various cancers, and there is ongoing research to identify specific inhibitors that could improve patient outcomes [190]. Other members of the lysyl oxidase family are also being considered as potential targets to mitigate collagen fibrosis [191,192].

Stimulation of endogenous matrix proteases, which naturally degrade collagen, or the application of exogenous collagenases remains a viable strategy for removing excessive collagen deposits. Developing targeted therapies may offer greater success compared to previously explored systemic approaches [193].

#### Tuning collagen biosynthesis and folding machinery.

The abnormal quality of collagen folding and PTMs in genetic diseases can potentially be improved by targeting collagen biosynthesis and folding mechanisms. A recent cell culture study using exogenous recombinant HSP47, which was taken up by the cells, demonstrated increased collagen secretion, reduced collagen overmodification, and decreased intracellular collagen retention in an *osteogenesis imperfecta* model [194]. Missense mutations within collagen sequences, particularly glycine substitutions, can delay the zipper-like folding of the triple helix. As a result, the unfolded chains remain accessible to the PTM machinery for a longer period and may accumulate excessive modifications [50,51]. To address the issue of overmodification, two strategies can be implemented: 1) Pharmacological chaperones that help restart the folding of the triple helix beyond the mutation site, minimizing the delay in the folding process [1]; and 2) A limited reduction in the enzymatic activities of the collagen PTM machinery. The practical application of both strategies still requires further research and development.

## Conclusions

The collagen triple helix is a crucial and abundant structure found in more than 80 different proteins in our body. It is associated with various genetic and acquired diseases. Its complexities remain somewhat mysterious, and there is still much to uncover in the quest for effective therapies. The development of new tools to study, diagnose, and treat diseases related to the collagen triple helix should motivate both basic and clinical researchers to more effectively meet our health needs.

## Supplementary Material

1

2

Supplementary material associated with this article can be found, in the online version, at doi:10.1016/j.matbio.2025.07.003.

This article contains supplementary Figures S1 and S2.

## Figures and Tables

**Table:** **Collagen-like proteins** (in alphabetical order).

Name	UniProt identifier	Class
Acetylcholinesterase collagenic tail peptide	Q9Y215	Miscellaneous
Adiponectin	Q15848	Bouquet-like (C1q subclass)
Collagen and calcium-binding EGF domain-containing protein 1	Q6UXH8	Miscellaneous
Collagen triple helix repeat-containing protein 1	Q96CG8	Miscellaneous
Collectin-10	Q9Y6Z7	Bouquet-like (C-type lectin subclass)
Collectin-11	Q9BWP8	Bouquet-like (C-type lectin subclass)
Collectin-12	Q5KU26	Transmembrane
Complement C1q subcomponent subunit A	P02745	Bouquet-like (C1q subclass)
Complement C1q subcomponent subunit B	P02746	Bouquet-like (C1q subclass)
Complement C1q subcomponent subunit C	P02747	Bouquet-like (C1q subclass)
Complement C1q-like protein 2	Q7Z5L3	Bouquet-like (C1q subclass)
Complement C1q-like protein 3	Q5VWW1	Bouquet-like (C1q subclass)
Complement C1q-like protein 4	Q86Z23	Bouquet-like (C1q subclass)
Complement C1q tumor necrosis factor-related protein 1	Q9BXJ1	Bouquet-like (C1q subclass)
Complement C1q tumor necrosis factor-related protein 2	Q9BXJ5	Bouquet-like (C1q subclass)
Complement C1q tumor necrosis factor-related protein 3	Q9BXJ4	Bouquet-like (C1q subclass)
Complement C1q tumor necrosis factor-related protein 5	Q9BXJ0	Bouquet-like (C1q subclass)
Complement C1q tumor necrosis factor-related protein 6	Q9BXI9	Bouquet-like (C1q subclass)
Complement C1q tumor necrosis factor-related protein 7	Q9BXJ2	Bouquet-like (C1q subclass)
Complement C1q tumor necrosis factor-related protein 8	P60827	Bouquet-like (C1q subclass)
Complement C1q and tumor necrosis factor-related protein 9A	P0C862	Bouquet-like (C1q subclass)
Complement C1q and tumor necrosis factor-related protein 9B	B2RNN3	Bouquet-like (C1q subclass)
C1q-related factor	O75973	Bouquet-like (C1q subclass)
Ectodysplasin-A	Q92838	Transmembrane
EMILIN-1	Q9Y6C2	Miscellaneous
EMI domain-containing protein 1	Q96A84	Miscellaneous
Ficolin-1	O00602	Paw-like
Ficolin-2	Q15485	Paw-like
Ficolin-3	O75636	Paw-like
Gliomedin	Q6ZMI3	Transmembrane
Macrophage receptor MARCO	Q9UEW3	Transmembrane
Macrophage scavenger receptor types I and II	P21757	Transmembrane
Mannose-binding protein	P11226	Bouquet-like (C-type lectin subclass)
Otolin-1	A6NHN0	Miscellaneous
Pulmonary surfactant-associated protein A1	Q8IWL2	Bouquet-like (C-type lectin subclass)
Pulmonary surfactant-associated protein A2	Q8IWL1	Bouquet-like (C-type lectin subclass)
Pulmonary surfactant-associated protein D	P35247	Cruciform + Fuzziball
Scavenger receptor class A member 3	Q6AZY7	Transmembrane
Scavenger receptor class A member 5	Q6ZMJ2	Transmembrane

## Data Availability

Data will be made available on request.

## Abbreviations:

AGE: advanced glycation end products
CBD: collagen-binding domain
CHP: collagen hybridizing peptide
CMP: collagen mimetic peptide
CNA35: 35 kDa fragment of collagen adhesin from *Staphylococcus aureus*
HSP47: heat-shock protein 47
Hyl: hydroxylysine
Hyp: hydroxyproline
LOXL2: lysyl oxidase-like2
PTM: post-translational modification
TH: triple helix
THP: triple helical peptide
TM: transmembrane
