# Supplementary material for: Around the collagen triple helix: an introduction to studying associated genetic and acquired diseases
Source: Matrix Biol. Author manuscript; Available in PMC 2026 May 4. (PMC13138384; doi:10.1016/j.matbio.2025.07.003)
Supplement: 1 [file NIHMS2169391-supplement-1.pdf]

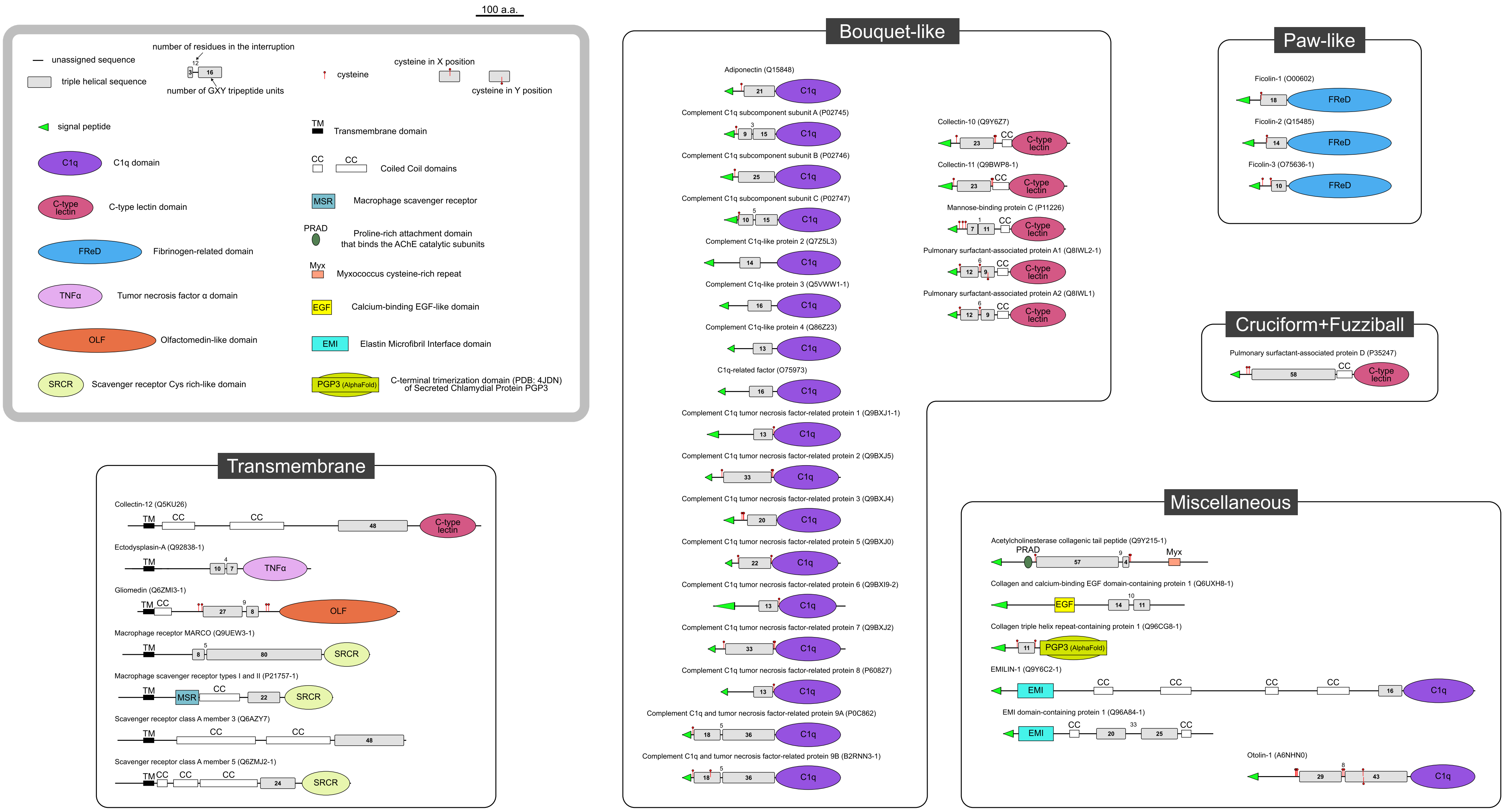

100 a.a.
unassigned sequence
triple helical sequence
number of residues in the interruption
number of GXY tripeptide units
cysteine
cysteine in X position
cysteine in Y position
signal peptide
C1q domain
C-type lectin domain
Fibrinogen-related domain
Tumor necrosis factor α domain
Olfactomedin-like domain
Scavenger receptor Cys rich-like domain
Transmembrane domain
Coiled Coil domains
Macrophage scavenger receptor
Proline-rich attachment domain that binds the AChE catalytic subunits
Myxococcus cysteine-rich repeat
Calcium-binding EGF-like domain
Elastin Microfibril Interface domain
C-terminal trimerization domain (PDB: 4JDN) of Secreted Chlamydial Protein PGP3
Bouquet-like
Adiponectin (Q15848)
Complement C1q subcomponent subunit A (P02745)
Complement C1q subcomponent subunit B (P02746)
Complement C1q subcomponent subunit C (P02747)
Complement C1q-like protein 2 (Q7Z5L3)
Complement C1q-like protein 3 (Q5VWW1-1)
Complement C1q-like protein 4 (Q86Z23)
C1q-related factor (O75973)
Complement C1q tumor necrosis factor-related protein 1 (Q9BXJ1-1)
Complement C1q tumor necrosis factor-related protein 2 (Q9BXJ5)
Complement C1q tumor necrosis factor-related protein 3 (Q9BXJ4)
Complement C1q tumor necrosis factor-related protein 5 (Q9BXJ0)
Complement C1q tumor necrosis factor-related protein 6 (Q9BXI9-2)
Complement C1q tumor necrosis factor-related protein 7 (Q9BXJ2)
Complement C1q tumor necrosis factor-related protein 8 (P60827)
Complement C1q and tumor necrosis factor-related protein 9A (P0C862)
Complement C1q and tumor necrosis factor-related protein 9B (B2RNN3-1)
Collectin-10 (Q9Y6Z7)
Collectin-11 (Q9BWP8-1)
Mannose-binding protein C (P11226)
Pulmonary surfactant-associated protein A1 (Q8IWL2-1)
Pulmonary surfactant-associated protein A2 (Q8IWL1)
Paw-like
Ficolin-1 (O00602)
Ficolin-2 (Q15485)
Ficolin-3 (O75636-1)
Cruciform+Fuzziball
Pulmonary surfactant-associated protein D (P35247)
Transmembrane
Collectin-12 (Q5KU26)
Ectodysplasin-A (Q92838-1)
Gliomedin (Q6ZMI3-1)
Macrophage receptor MARCO (Q9UEW3-1)
Macrophage scavenger receptor types I and II (P21757-1)
Scavenger receptor class A member 3 (Q6AZY7)
Scavenger receptor class A member 5 (Q6ZMJ2-1)
Miscellaneous
Acetylcholinesterase collagenic tail peptide (Q9Y215-1)
Collagen and calcium-binding EGF domain-containing protein 1 (Q6UXH8-1)
Collagen triple helix repeat-containing protein 1 (Q96CG8-1)
EMILIN-1 (Q9Y6C2-1)
EMI domain-containing protein 1 (Q96A84-1)
Otolin-1 (A6NHN0)
