# Supplementary figures and images for: Around the collagen triple helix: an introduction to studying associated genetic and acquired diseases
Source: Matrix Biol. Author manuscript; Available in PMC 2026 May 4. (PMC13138384; doi:10.1016/j.matbio.2025.07.003)

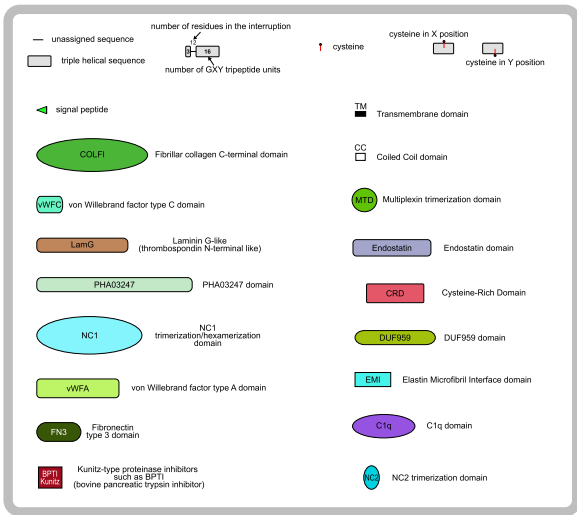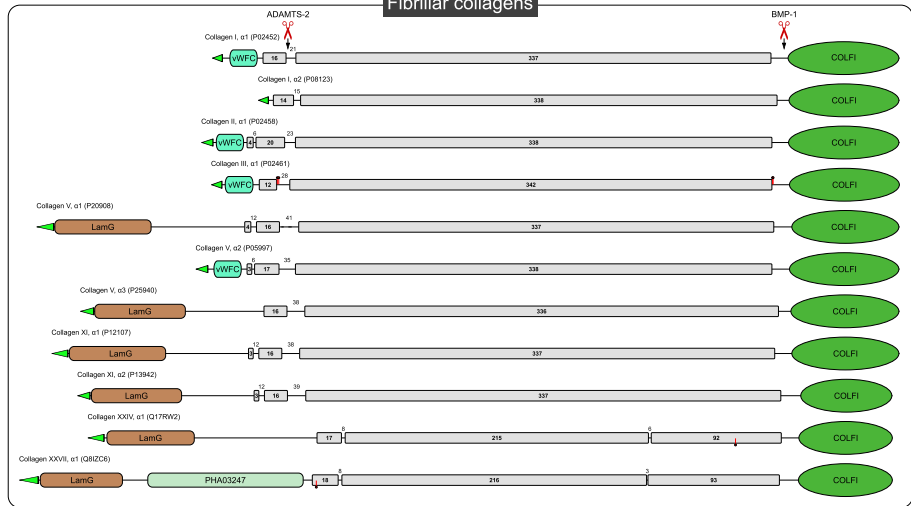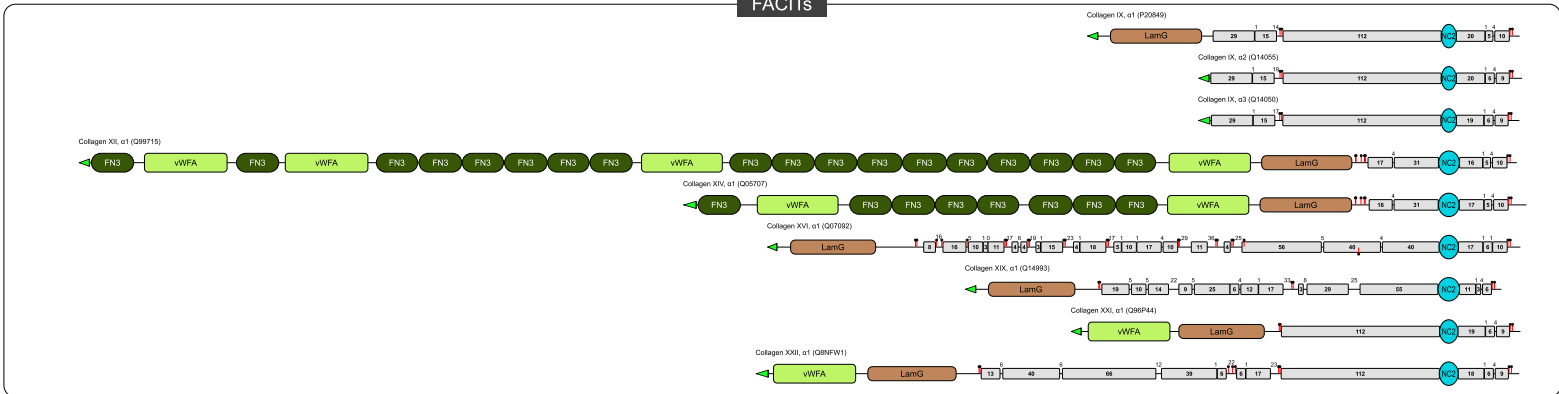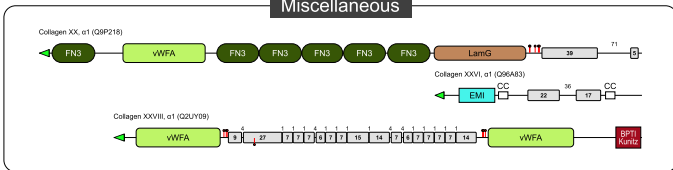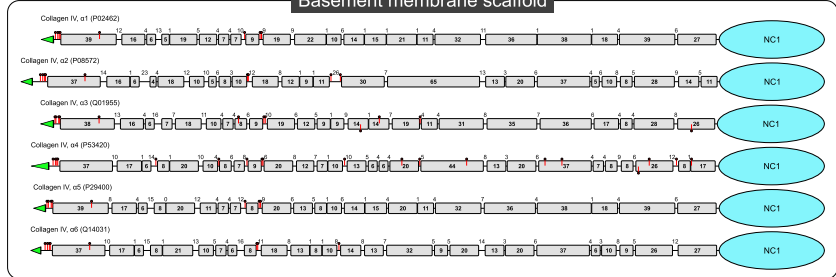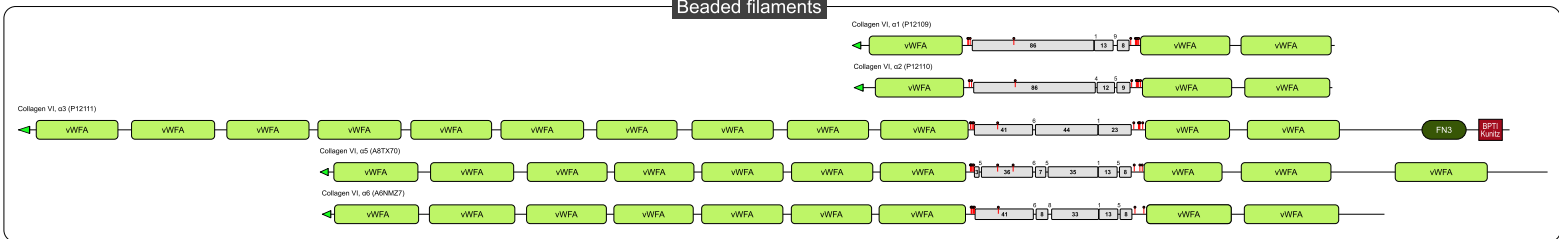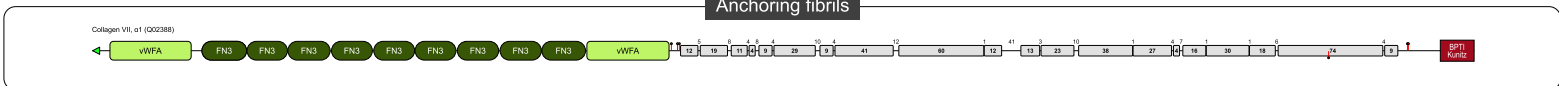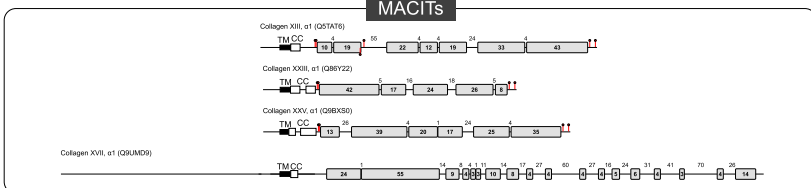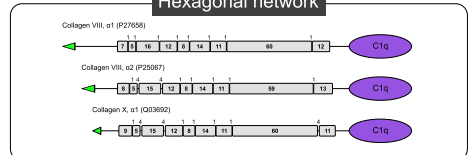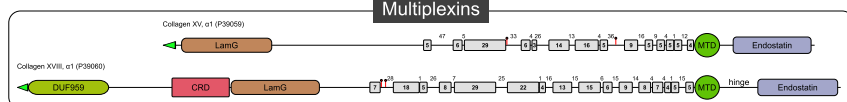

Supplement: 2 [file NIHMS2169391-supplement-2.pdf]
